# Electroacupuncture as an effective therapy for Tapia’s syndrome after transoral intubation for general anesthesia: a case report and review of the literature

**DOI:** 10.1186/s13256-022-03632-z

**Published:** 2022-11-05

**Authors:** Luan Xie, Zhenyu Xiong, Weihong Xiao, Yingnan Mo, Xiangliang Li, Xuan Zhuang, Ying Yang, Haipeng Jin

**Affiliations:** 1 College of Acupuncture, Fujian University of Traditional Chinese Medicine, Fuzhou, 350122 China; 2 Department of Rehabilitation, Xiamen Hospital of Beijing University of Chinese Medicine, Xiamen, 361000 China

**Keywords:** Tapia’s syndrome, Electroacupuncture, Dysphagia, Dysarthria, Case report, Swallowing

## Abstract

**Background:**

Tapia’s syndrome is a rare complication of airway manipulation under general anesthesia. Injuries to the vagus nerve (X) and hypoglossal nerve (XII) during transoral intubation are the primary cause of the disease. The typical symptoms include hoarseness, dysarthria, dysphagia, tongue muscle atrophy, and tongue deviation toward the affected side. We report a case of Tapia’s syndrome treated with electroacupuncture to accelerate the recovery process, and discuss the potential mechanism behind our findings based on previous research.

**Case presentation:**

In this report, we describe a 57-year-old Chinese man who suffered Tapia’s syndrome after craniotomy evacuation of hematoma with general anesthesia and transoral intubation. After 52 days of electroacupuncture therapy along with standard swallowing training, the patient achieved significant improvement in deglutition and speech function.

**Conclusion:**

Electroacupuncture is effective and safe for Tapia’s syndrome. It can shorten the recovery time when combined with routine swallowing rehabilitation.

## Background

Tapia’s syndrome, which was first described in 1904 by a Spanish otorhinolaryngologist named Antonio Garcia Tapia, is a rare complication of airway manipulation caused by transoral intubation. The fundamental cause of the syndrome is an injury to the cranial nerves, to be specific, damage to the vagus nerve (X) and hypoglossal nerve (XII) caused by the stretching and compression during transoral intubation [1]. The characteristic symptoms of Tapia’s syndrome are related to palsy of cranial nerves X and XII, and include hoarseness, dysarthria, dysphagia, tongue muscle atrophy, and trouble with tongue movement [2]. In reviewing the literature, we found that more than half of the patients have clinical histories of rhinoplasty or septorhinoplasty, and that transoral intubation was present in most of these cases [3]. Therefore, patients who receive transoral intubation during surgical operations are likely to suffer from Tapia’s syndrome. It is generally acknowledged that palsy of nerves is temporary, and the injured nerves can recover gradually. However, without active intervention, complications such as aspiration pneumonia and malnutrition are more likely to occur, thus delaying the progress of recovery. There is no established treatment regimen for Tapia’s syndrome, and commonly used treatment consists of medication and rehabilitation: a short course of corticosteroid (pattern 1: intravenous prednisolone 20 mg/day for 3 days, oral corticosteroid 5 mg/days for 10 days; pattern 2: oral prednisolone 60 mg/day, tapered down over a period of 14 days) and vitamin B complex is applied as conventional medication [4], and standard swallowing training is used as the main method of rehabilitation [5]. Even with these positive interventions, it generally takes up to 4–6 months to regain nerve function [6].

In recent years, electroacupuncture has demonstrated a favorable effect on the recovery from peripheral nerve injury (PNI), for instance, Bell’s palsy [7], diabetic peripheral neuropathy [8], and chemotherapy-induced peripheral neuropathy [9], and so on. However, there has been no reported application of electroacupuncture therapy in any studies related to Tapia’s syndrome before. In our study, apart from routine swallowing rehabilitation treatment, the patient also received electroacupuncture therapy, and surprisingly, his recovery process shortened to 52 days.

## Case presentation

A 57-year-old Chinese man who suffered from spontaneous subarachnoid hemorrhage following the rupture of a cerebral aneurysm underwent craniotomy evacuation of hematoma with general anesthesia and transoral intubation. No problems with swallowing or speaking were reported during the surgery. Unfortunately, the patient experienced dysarthria, dysphagia, and trouble with tongue movement after the surgery.

**Fig. 1:**
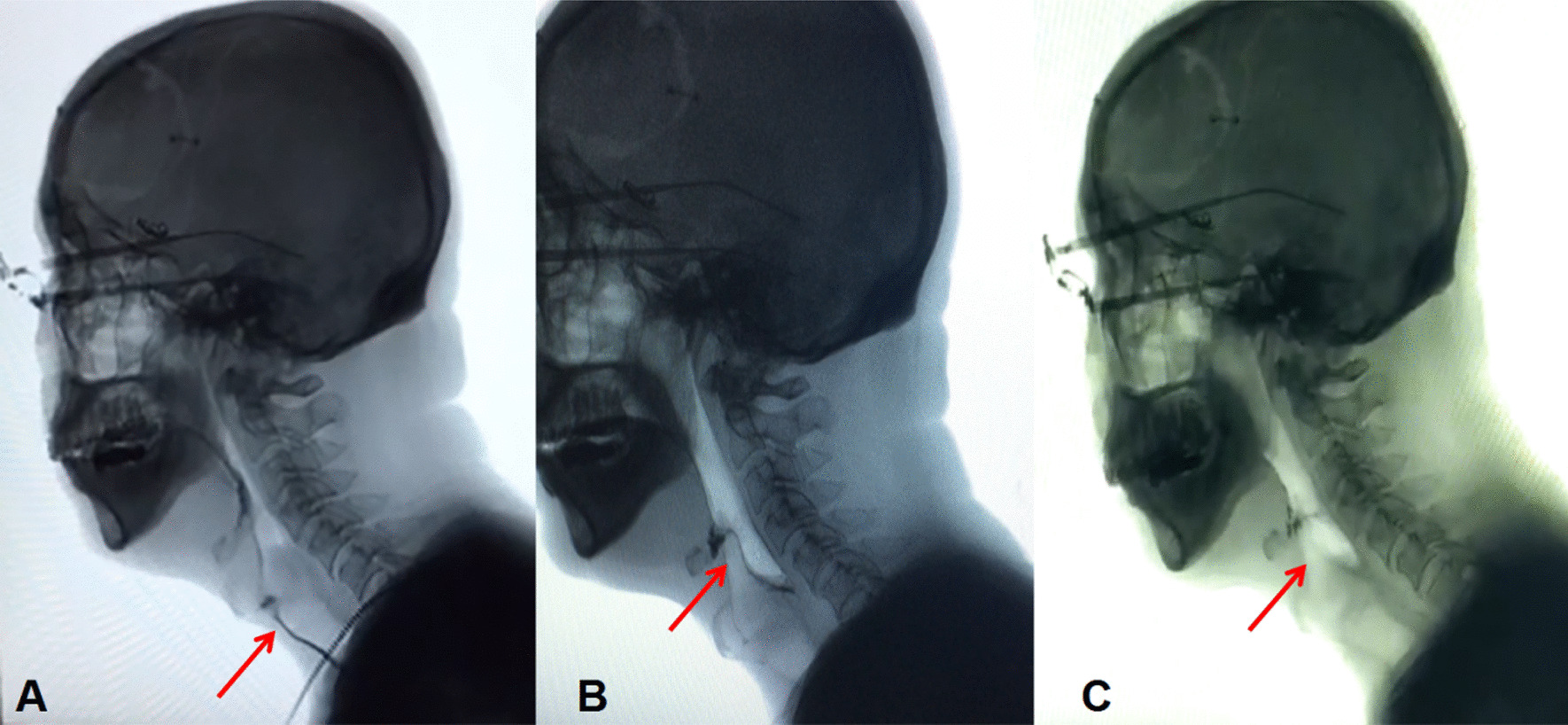
Videofluoroscopic swallowing study (VFSS). **A** Aspiration occurred when the patient drank 3 ml of thin liquid. (before treatment, arrow, left). **B** A small amount of leakage when the patient drank 3 ml of thin liquid. (after 2 weeks of treatment, arrow, middle). **C** No leakage or aspiration occurred when the patient drank 3 ml of thin liquid. (after 52 days of treatment, arrow, right)

**Fig. 2:**
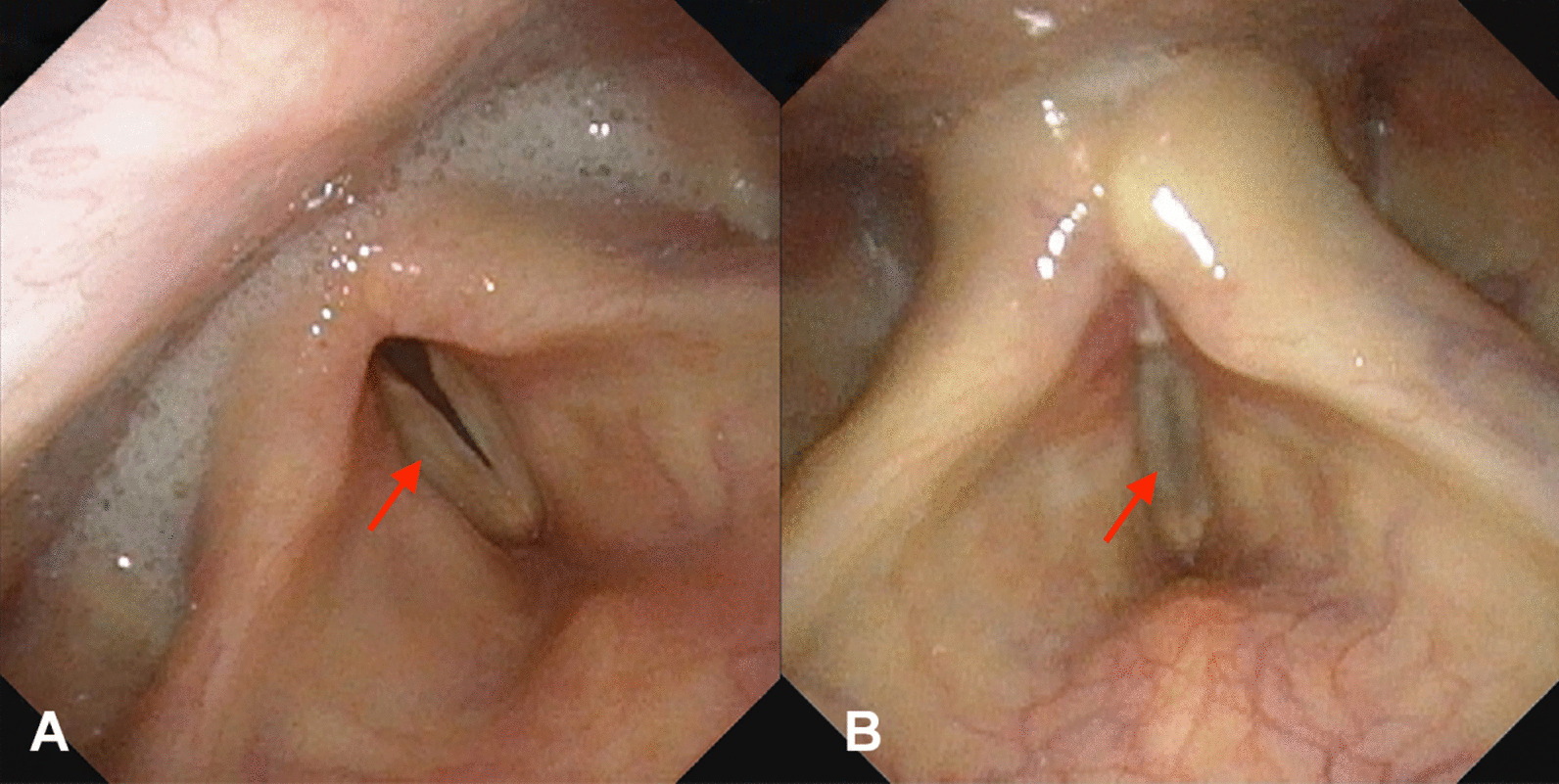
The electronic laryngoscope. **A** Paralysis of bilateral vocal folds (before treatment, arrow, left). **B** Vocal folds could close up (after treatment, arrow, right)

**Fig. 3:**
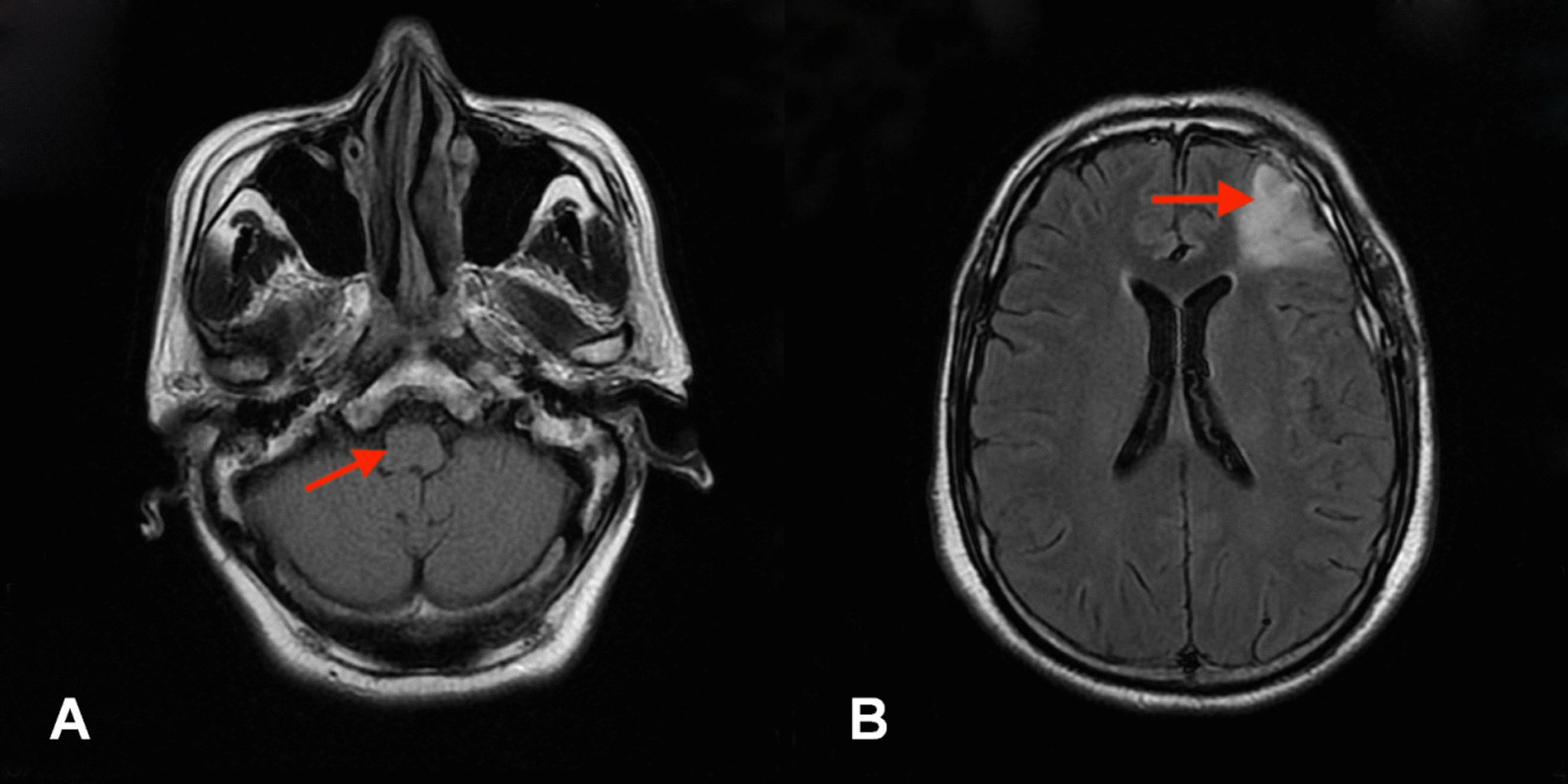
Magnetic resonance imaging. **A** No lesion concerning the brainstem (arrow, left). **B** Symptoms cannot be explained by left frontal lobe malacia and subdural hematoma (arrow, right)

Thus, we performed a detailed neurological examination. In addition to the symptoms described above, we also found symptoms linked to cranial nerve injuries, such as a weakened pharyngeal reflex, tongue deviation toward the affected side, atrophy of tongue muscle, and so on. Further evaluations and imaging examinations were carried out. The rating of the functional oral intake scale (FOIS) was level 2 [10]. Videofluoroscopic swallowing study (VFSS) showed that the contrast agent leaked into the vestibule of the larynx and entered the trachea through the glottis when the patient drank 3 ml of thin liquid (Fig. 1A). The rating of Rosenbek leakage/aspiration scale was level 8, which is the worst level. When the patient drank 3 ml of medium-thick semi-fluid liquid, effective swallowing was not initiated and the epiglottis valley had a large amount of residue. When examined through the electronic laryngoscope, we noticed paralysis of bilateral vocal folds (Fig. 2A). All these observations indicated that the vagus nerve and hypoglossal nerve had been injured. Therefore, we thought that there may be a lesion in or near the brainstem that led to nerve injury. However, magnetic resonance imaging (MRI) showed that there were no brainstem lesions (Fig. 3A), and that the symptoms that arose after surgery could not be explained by the left frontal lobe malacia and subdural hematoma (Fig. 3B). Owing to the inconformity of symptoms and radiographic results, we decided to analyze the rare case again. When we reviewed the clinical history, we noticed that the surgery was performed under general anesthesia and transoral intubation, so there may be a connection between transoral intubation and nerve injury. After consulting with the ear–nose–throat (ENT) and anesthesiology departments, we excluded arytenoid dislocation and violent intubation. Then we found that these symptoms are similar to those of Tapia’s syndrome, so we compared the characteristics of this case with diagnostic criteria of Tapia’s syndrome. First, in terms of clinical history, the majority of Tapia’s syndrome cases occur following the surgical intervention under general anesthesia and transoral intubation. Our patient went through craniotomy evacuation of hematoma with general anesthesia and transoral intubation before these symptoms arose. Second, symptoms of the patient included difficulties in tongue movement, unilateral tongue paralysis, and atrophy of the tongue, as well as hoarseness and dysphagia, which matches the described symptoms of Tapia’s syndrome. Third, the complete neurological examination showed that there are cranial nerve injuries and, based on the MRI results, we excluded lesions in the brainstem. Hence the symptoms are likely caused by peripheral nerve injuries. Thus, a diagnosis of Tapia’s syndrome was made.

We designed a detailed rehabilitation strategy for the patient immediately. In the previous studies of Tapia’s syndrome, only standard swallowing training was used as rehabilitation treatment, which proved to be effective but still means a lengthy recovery process. So we decided to include electroacupuncture as a measure in our treatment to observe the effect.

**Fig. 4:**
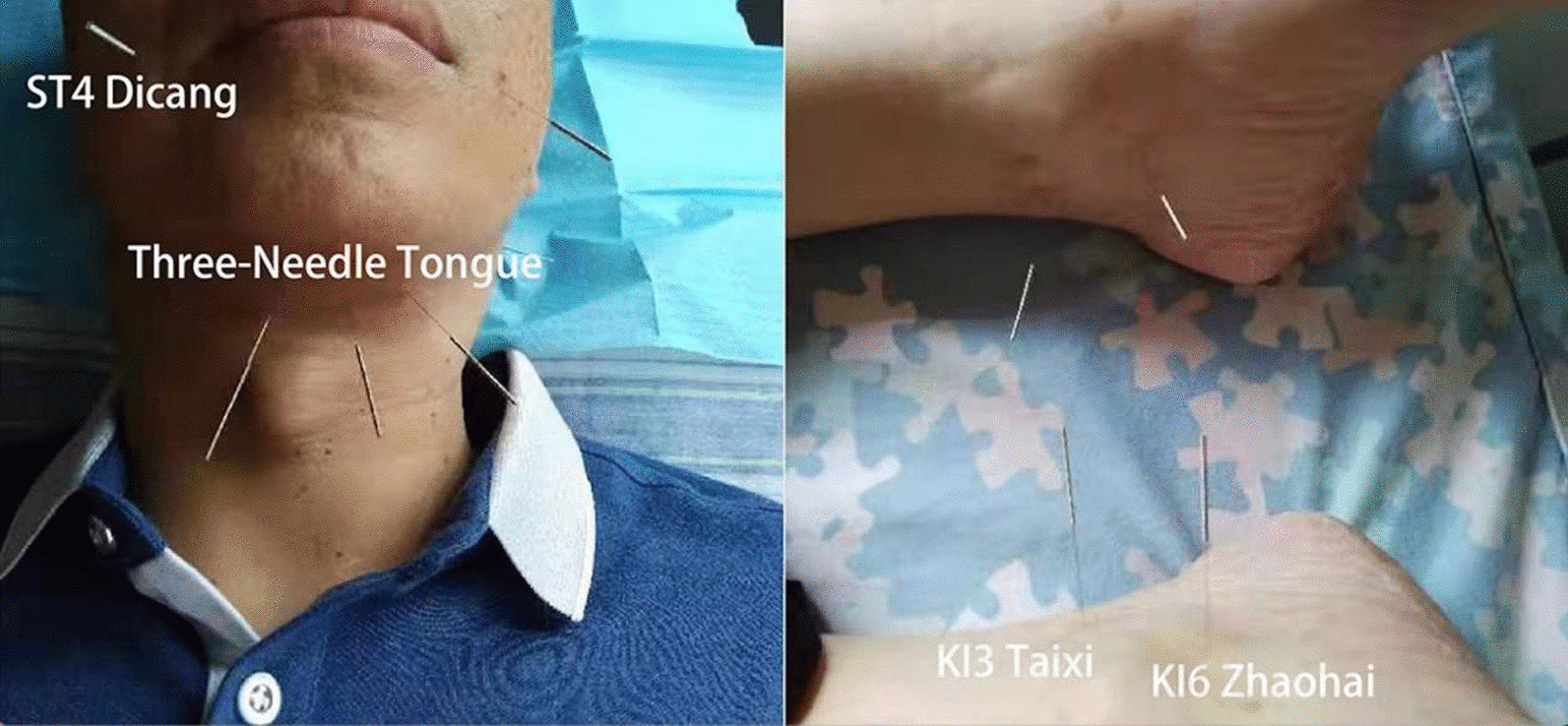
The location of acupoints. Acupoints on face and neck (three-needle tongue and Dicang). Acupoints on lower limbs (Taixi and Zhaohai)

The patient received acupuncture treatment 30 minutes per day, six times per week for 52 days (Fig. 4). Qi She brand disposable, sterile steel needles (0.25 × 40 mm; Wuxi Jiajian Medical Instrument Co. Ltd) were used. Based on our experience in treating similar diseases, we chose several acupoints to improve deglutition and speech function. These acupoints included the three-needle tongue (Shanglianquan, located in the depression between the lingual bone and the border of the lower jaw, 1 cun inferior to the midline of the jaw), and two other acupoints located at 0.8 cun bilateral to Shanglianquan (note that the width of the interphalangeal joint of the patient’s thumb is taken as 1 cun, approximately 25 mm), Dicang (ST 4, with the eyes looking straight forward, the point is vertically below the pupil, at the level of the angle of the mouth), Taixi (KI 3, in the depression between the tip of the medial malleolus and Achilles’ tendon), and Zhaohai (KI 6, in the depression below the tip of the medial malleolus). Electroacupuncture (EA) stimulation (EA parameters were set as follows: discontinuous wave with frequency 5 Hz, pulse width 1 ms, and intensity approximately 2 mA, until a slight jitter in the muscle is observed) was applied to two acupoints located at 0.8 cun bilateral to Shanglianquan (EX). No side effects such as bleeding or hematoma were observed during the entire treatment.

We observed gradual improvement in the patient’s deglutition and speech function throughout the treatment, and after 52 days of the treatment he had fully recovered: the patient could swallow and drink water normally without coughing and there was an improvement in his speech function. The FOIS rating after treatment was level 6, a significant improvement compared with the level 2 before treatment. As for VFSS, after 2 weeks of treatment, there was a small amount of leakage when the patient drank 3 ml of thin liquid (Fig. 1B). The rating of Rosenbek leakage/aspiration scale was level 4. After 52 days of treatment, the patient drank 3 ml of fluid without leakage or aspiration (Fig. 1C) and the rating of Rosenbek leakage/aspiration scale was level 2. While the patient drank 10 ml of medium-thick semi-fluid liquid, there was less residue in the epiglottis valley, which could be cleared after repeated swallowing and coughing. No aspiration occurred during the entire process either. The bilateral vocal folds paralysis has been resolved so the vocal folds can close up, which means better swallow function and sound control (Fig. 2B). These evaluations and examinations illustrated that the patient’s swallowing function had basically recovered and the risk of silent aspiration was greatly reduced. Therefore, after 52 days of treatment, the patient’s nasal feeding tube was successfully removed.

## Discussion

Patients who experience Tapia’s syndrome are likely to have a clinical history of surgical intervention. Common surgical interventions include rhinoplasty or septorhinoplasty, intracranial surgery, and fracture repair surgery. Most patients with Tapia’s syndrome have clinical histories of transoral intubation [11]. Stretching and compression during the transoral intubation may damage the vagus (X) and hypoglossal (XII) cranial nerves [12]. Additionally, Tapia’s syndrome can be considered a localized lesion at the crossing of the recurrent laryngeal and hypoglossal nerves. It has been suggested that pressure neuropathy occurs as a result of hyperinflation or malposition of the cuff of the endotracheal tube within the larynx, causing compression of both nerves at this crossing point [13]. Dysphagia is the biggest challenge for patients with Tapia’s syndrome, it not only affects the intake of water and food, but also causes aspiration pneumonia and delays the postoperative recovery process [14, 15]. In terms of treatment, people usually combine medication with rehabilitation at the same time [16, 17]. Corticosteroids and vitamin B complex are most widely used, but swallowing and speech therapy play a more prominent role during the treatment [18].

After assessing the swallowing function of the patient, we summarized the main problems concerning this case. First, weakness of tongue movement and a decrease in larynx elevation meant that it was hard to enable food to enter the esophagus from the mouth. Second, the weakened pharyngeal and cough reflex increased the risk of aspiration, which might lead to pneumonia. Food remained in the epiglottis valley and pyriform sinus after repeated swallowing. Apart from standard swallowing training, we intended to include electroacupuncture in the treatment to enhance the therapeutic effect.

Numerous studies [19, 20] have demonstrated that electroacupuncture facilitates the recovery from dysphagia, dysarthria, and tongue muscle atrophy, although no studies have reported the use of electroacupuncture as a treatment for Tapia’s syndrome. In this case study, we designed our electroacupuncture treatment plan based on our expertise in electroacupuncture to resolve the dysphagia problem for our patient with Tapia’s syndrome.

In traditional Chinese medicine theory, dysphagia is caused by the blockage of *qi* and blood in the channel of throat and tongue. *Qi* and blood are regarded as energy promoting growth and development of the body, and the channel is responsible for transferring *qi* and blood to all organs and tissues. Transoral intubation caused damage to the channel in the throat, *qi* and blood failed to reach the throat and tongue, and as a consequence, the patient could not swallow as normal. In addition, electroacupuncture has the advantage of dredging the channel and regulating *qi* and blood. Stimulating three-needle tongue enhances the circulation of *qi* and blood around the throat. On the basis of the theory of local and nearby therapeutic effect, stimulation at Dicang (ST 4) is an effective therapy for improving swallowing. Although Taixi (KI 3) and Zhaohai (KI 6) sites are in the lower limb, these two acupoints belong to the circulation of kidney channels. This kidney branch can reach the neck, throat, and both sides of the tongue, so they have marked effectiveness on dysphagia. Treatment with Zhaohai is effective in improving deglutition according to the traditional Chinese medicine theory of eight confluent points.

Most studies paid close attention to the effect and mechanism of electroacupuncture therapy on dysphagia after stroke. Few focused on dysphagia caused by peripheral nerve injury such as Tapia’s syndrome. Swallowing is a complicated process: numerous parts of the nervous system, especially the brainstem, thalamus, basal ganglia, cranial nerves, motor, and sensory cortices, are involved. The whole process requires the activation of 55 individual muscles and six relevant cranial nerves [21]. Our opinion is that the effectiveness of electroacupuncture depends mainly on the acupoints it stimulates, while the function of acupoints are related to their anatomical position. Several acupoints were selected to improve swallowing function because of the abundant nerves in this position, including important nerves to control a series of movements of swallowing, such as the glossopharyngeal, vagus and hypoglossal nerves. Injuries of the vagus and hypoglossal nerves are the direct cause of Tapia’s syndrome. In the lower limb, these two acupoints belong to the circulation of kidney channels. This kidney branch can reach the neck, throat, and both sides of the tongue. Chen *et al.* [22] found that after a conventional 6-week acupuncture treatment, rats exhibited a more mature ultrastructural nerve organization, with significantly higher axon density, blood vessel area, and percentage of blood vessel area occupied in the total nerve area, which meant acupuncture treatment could have positive effects on the regeneration of dissected nerves. Electroacupuncture could combine both the needling and electrical stimulation to potentiate the effect of the acupuncture treatment. Previous studies of nerve injuries in the limbs of rats conducted by Hoang *et al.* [23] showed that acupuncture and electroacupuncture could accelerate the maturation of regenerated nerves with larger mean values of axon number, total nerve area, blood vessel number, blood vessel area, and nerve amplitude. These studies have shown that acupuncture and electroacupuncture appear to have a positive effect on the regeneration process. Liu *et al.* [24] found that electroacupuncture promoted sciatic nerve function recovery through downregulating miR-1b after PNI. Motohiro *et al.* [25] indicated that electroacupuncture with distal cathode orientation accelerated the elongation of regenerating axons in the direction of the target tissue (interosseous, lumbrical, and flexor digitorum brevis muscles), and enhanced the functional contact of the nerve terminal with the motor endplate. Besides, reduction of the atrophy and degeneration of the target organs will be beneficial to the recovery of nerve function. Acupuncture accelerated the reflex time of random tongue movement and increased the coordination of muscle movement related to swallowing, promoted movement of the tongue and masticatory muscle, improved blood flow in the pharynx and larynx, and alleviated muscle atrophy [26]. In this case, we observed that electroacupuncture improved swallowing function and reduced the recovery time from 6 months to 52 days for a patient with Tapia’s syndrome. This is the first reported case of electroacupuncture treatment for Tapia’s syndrome. We believe that the general mechanism discussed above explains the positive effect of electroacupuncture on Tapia’s syndrome. This patient should be characterized as a severe case of Tapia’s syndrome (Grade III), since he exhibited difficulties in feeding and drinking. It usually takes 4–6 months of treatment to achieve recovery [27–29]. We believe that the electroacupuncture treatment in our case accelerated the recovery process for this patient.

## Conclusion

Although the incidence of Tapia’s syndrome is low, we still need to pay more attention to it. Our study illustrates that electroacupuncture therapy combined with standard swallowing training is beneficial to enhance the patient’s recovery. Furthermore, it is important to note that we used no corticosteroids in this case and still achieved a remarkable effect. That is to say, electroacupuncture therapy is an optional method for those patients with contraindications for corticosteroids. We will carry on further research to provide a more powerful theoretical basis for electroacupuncture treatment of Tapia’s syndrome.

## Data Availability

Not applicable.

## Abbreviations

VFSS: Videofluoroscopic swallowing study
FOIS: Functional oral intake scale
ENT: Ear–nose–throat department
PNI: Peripheral nerve injury
EA: Electroacupuncture
SC: Schwann cell
